# The New Frontier of Esports and Gaming: A Scoping Meta-Review of Health Impacts and Research Agenda

**DOI:** 10.3389/fspor.2021.640362

**Published:** 2021-03-16

**Authors:** Sarah Kelly, Janni Leung

**Affiliations:** ^1^UQ Business School, University of Queensland, Brisbane, QLD, Australia; ^2^School of Psychology, University of Queensland, Brisbane, QLD, Australia

**Keywords:** video gaming, eSports, health, meta-review, well-being

## Abstract

Given the rapid evolution of the gaming industry and the rising popularity of a hyper-connected, competitive esports version of online gaming, a meta-review of the impact of online competitive gaming upon health is timely. A scoping meta-review was conducted on 10 reviews that reported on any health consequences (physical, lifestyle, cognitive, mental, or social) of esports, online competitive gaming, or video gaming participation, as a player or spectator. While past reviews have examined health effects of video gaming, few have focused upon the newly evolved gaming context, incorporating both playing and streamed viewing, recognition as a professional sport, and potential career and exponential participation. Most past reviews have focused upon physical health impacts of video gaming among adolescents and young adults, but none have examined impacts of different forms of gaming participation in the new gaming era, and their potential differential health impacts. A scoping meta-review was undertaken on the physical, social, and psychological health outcomes of competitive online gaming and associated screen use, revealing a need for further review and research into lifestyle health outcomes including diet and sedentary behavior among young esports and competitive video gaming participants.

## Introduction

Video gaming over the past two decades has evolved into a hyper-connected, highly commercialized, competitive system of online gaming. The exponential growth and commercialization of “esports,” which is an umbrella term encompassing different tournaments, competitions, events, and games, signals a mainstream, global phenomenon among millennial and Gen Z consumers. Esports is quickly becoming one of the world's largest entertainment industries, with a net worth exceeding 650 million US dollars in 2017 and estimated to increase to 1.5 billion US dollars by 2020 (Gough, 2020). The global audience for esports will reach approximately 600 million in 2023 (Tran, 2020), predominantly via platforms such as Twitch and YouTube. Esports participants (including both players and spectators) are largely young, well-educated males from high socio-economic backgrounds, making esports an attractive platform for luxury, and non-endemic sponsors (Hallmann and Giel, 2018). Sports including the NFL, NBA, Formula One, FIFA, AFL, and EPL are also diversifying into esports as a means to ensure their brands remain relevant to future generations of consumers. Esports is now increasingly being recognized as a sport, with an International Olympic sub-committee recently approving esports as a sport for the 2022 Asian Games. However, its status is still subject to debate, with its nascent governance structures and commercially motivated core possibly outside the realm of established sports (Hallmann and Giel, 2018). Notwithstanding this debate, esports appears to embody many of the recognized attributes of sport including intense competition, significant participation and following, attraction of commercial sponsorship and prize money, and high performance training (Jenny et al., 2017).

Unlike traditional sports, the health benefits of esports are not immediately obvious (Ferguson, 2007; Boyle et al., 2011). Moreover, a growing body of research suggests that gaming may have a number of negative physical and mental health effects (Boyle et al., 2016; Taylor, 2018; Burleigh et al., 2019). “Gaming Disorder” has recently been listed as a mental disorder by the World Health Organization (World Health Organisation, 2020), whilst the American Psychiatric Association recognizes “Internet Gaming Disorder” as a potential diagnosis requiring further research (American Psychiatric Association, 2013). Given its increasing prevalence, determining the psychological and physiological effects of participation in online gaming, and how to appropriately protect against certain health risks, is paramount and has attracted the attention of policy makers, health practitioners, and the community alike.

We undertook a scoping meta-review on the physical, social, and psychological health impacts of competitive esports with the aim of identifying under-researched areas. We also sought to identify potential predictors of adverse outcomes of online gaming, particularly those associated with vulnerable populations, such as minors (Cook, 2020; Global Web Index, 2020).

Given the rapid evolution and rising popularity of esports, along with rising concerns regarding the lack of industry regulation (Griffiths and Nuyens, 2017; Funk et al., 2018), a meta-review of the health impacts of professional, competitive gaming as both a player and spectator sport is timely.

Our research, therefore, has a range of objectives:

1. To identify the physiological health impacts of participation in esports, including those related to physical health and cognitive function.
2. To identify the psychological health impacts of participation in esports, including mental health and social impacts.
3. To ascertain where there are gaps in the existing research and identify future research priorities.

## Method

We conducted a scoping meta-review of the extant empirical evidence on both the positive and negative health impacts of esports. Reviews were selected to synthesize the highest levels of evidence in the field (Aromataris et al., 2015) in order to facilitate an assessment of the quality of the evidence base and a summary and comparison of the reviews' findings (Smith et al., 2011).

The search was conducted of titles or abstracts in PubMed from 2011 for (esport^*^ OR video game^*^ OR video-game^*^ OR internet game^*^ OR online game^*^) and (a list of search terms covering the domains of physical health outcomes, health behaviors, mental health, psychological outcomes, cognitive outcomes, social outcomes, prosocial or antisocial behaviors, addiction, and gambling behaviors). Pubmed is a reliable and well- cited database for biomedical and clinical health-based studies and is also one of the largest and most detailed databases, with significant overlap with other health-focused databases (Ossom-Williamson and Minter, 2019). A supplementary search was conducted in Google Scholar using combinations of the following keywords: esports, esports rules, video games, gamers, professional gamers, gaming events, competitive video gaming, advertising, vloggers, forums, online communities, and virtual communities. If multiple reviews were conducted on the same health impact, the latest review with empirical data were included.

Data were collated from each selected review in relation to the definition of esports and the measured health impacts of esports. Results were synthesized narratively due to the anticipated heterogeneity of health impacts examined.

## Results

### Study Selection

**Figure 1:**
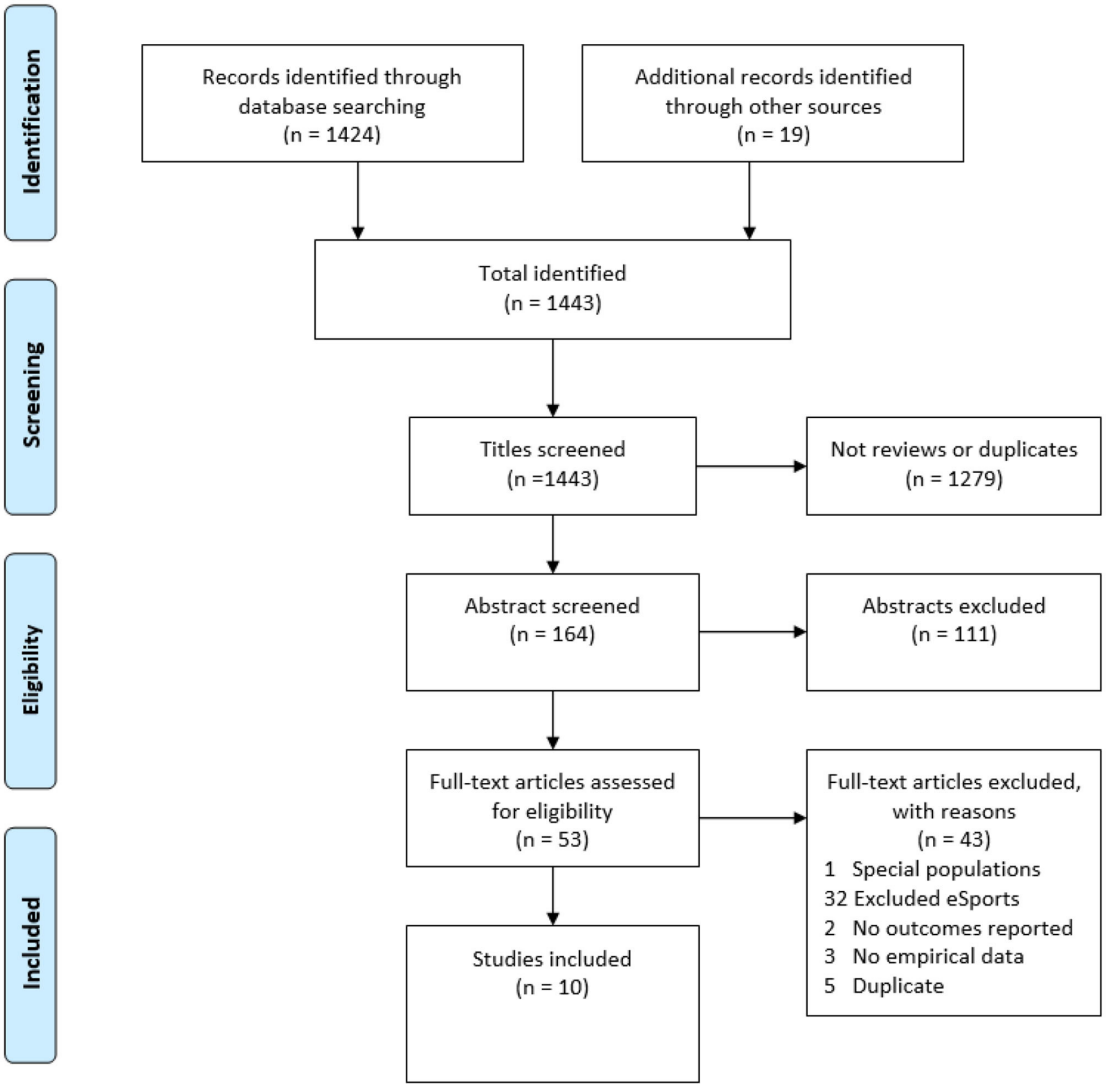
Flowchart presenting number of records identified, included, and excluded.

Using the PRISMA flowchart process shown in Figure 1, a total of 1,443 studies were identified. Title and abstract screening of those studies revealed 53 potentially eligible studies, from which 74% were excluded because the video gaming behavior studied did not include esports. Ten reviews, all of which examined a broad range of gaming behavior including esports, were included.

### Study Characteristics

The characteristics and findings of each examined review are set out in Tables 1, 2. Whilst a number of reviews did not focus on particular age groups, children and adolescents (e.g., age ≤ 18) were the most commonly studied group (see Table 1). The reviews were focused on internet gaming disorders (Mihara and Higuchi, 2017; Yao et al., 2017; González-Bueso et al., 2018; Paulus et al., 2018) or video gaming in general (Shams et al., 2015; Carson et al., 2016; Sala et al., 2018). One meta-analysis was conducted in relation to each of physiological and psychological outcomes (Gao et al., 2015), cognitive ability (Iannotti et al., 2009), and functional and structural neural alterations (Bilgrami et al., 2017). The smallest review included 24 studies (Gao et al., 2015), whilst the largest examined 252 (Paulus et al., 2018).

**Table 1:** Study characteristics of review on the impact of video gaming on health.

**Topic**	**Reference, base location**	**Study design and type of video game**	**Sample characteristics**	**Outcome measure assessed**
Physical outcomes	Gao et al., 2015, location of studies not reported	Meta-analysis of 35 papers published 1985–2015 on active video games	Children/adolescents < = 8; Study sample size > = 10	Physiological outcomes: energy expenditure, heart rate, VO2 max, metabolic equivalent (MET), physical activity, rate of perceived exertion, body composition and cardiovascular fitness
	Mihara and Higuchi, 2017, countries across Europe, North America, and Asia	Systematic review of 37 cross-sectional and 13 longitudinal studies on Internet gaming disorder published up to 2015	National representative samples, students, and online gamer samples	Physical pain
	Carson et al., 2016, 71 different countries	Systematic review of 235 studies published 2010–2014 on sedentary behavior and health indicators	School-aged children and youth, mean age: 5–17 years	Body composition Blood pressure Cardiometabolic health Cholesterol Cardiorespiratory fitness Muscular strength/endurance
	Bilgrami et al., 2017, location of studies not reported	Systematic review of 94 articles published 2006–2017	Adolescents engaged in new-age technologies including the internet, television, cell phones, and video games	Obesity
	Jalink et al., 2014, location of studies not reported	Systematic review of 38 articles (30 case reports, 7 case series, 1 prospective study) published up to 2014 on Nintendo video gaming systems	Any age	Injuries
Cognitive outcomes	Sala et al., 2018, location of studies not reported	Meta-analysis on 98 quasi-experimental studies papers comparing video game players and nonplayers conducted up to 2016	Age not specified; type of games included action video game (shooter and racing) players and nonaction video game players, mixed, and nonplayers	Cognitive ability measures: visual attention/procession, spatial ability, cognitive control, memory, intelligence/reasoning
	Shams et al., 2015, location of studies not reported	Systematic review of 27 prospective and 9 retrospective studies published 2011–2014 on video games	Various age groups and various samples e.g., casual gaming volunteers, online gaming addicts, pro-gamers	Cognition and brain structure
	Yao et al., 2017, location of studies not reported	Meta-analysis of 27 fMRI studies and 10 VBM studies published up to 2017 on Internet gaming disorders	Mean age 14–30; internet gaming disorder samples compared to healthy controls	Functional and structural neural alterations
Mental outcomes	Gao et al., 2015, location of studies not reported	Meta-analysis of 35 papers published 1985–2015 on active video games	Children/adolescents < = 18; Study sample size > = 10	Psychological outcomes: self-efficacy (toward AVGs/PA), enjoyment/liking, attitudes, intention, situational interest and intrinsic motivation
	Mihara and Higuchi, 2017, countries across Europe, North America, and Asia	Systematic review of 37 cross-sectional and 13 longitudinal studies on Internet gaming disorder published up to 2015	National representative samples, students, and online gamer samples	Internet gaming disorder Personality Psychological well-being Mental disorders and sleep
	Paulus et al., 2018, Australia, Austria, China, Germany, Iran, Netherlands, Norway, Singapore, South Korea, Spain, Taiwan, UK, USA, multi countries in Europe/Internationally	Systematic review of 252 articles published 1991–2016 on internet gaming disorder	Children and adolescents	Internet gaming disorder Psychological distress and reward seeking Mental disorders Self-esteem Self-efficacy
	González-Bueso et al., 2018, Australia, Austria, France, Finland, Norway, Germany, South Korea, Singapore, Spain, Sweden, Taiwan, UK, USA	Systematic review of 24 studies (21 cross-sectional, 3 prospective) published 2010–2017 on Internet gaming disorder	Any age; samples must be assessed by standardized questionnaires or criteria; internet addiction studies that specified that the internet was used to play videogames	Depression Anxiety ADHD/hyper-activity Social phobia/social anxiety
	Bilgrami et al., 2017, location of studies not reported	Systematic review of 94 articles published 2006–2017 on new-age technologies including the internet, television, cell phones, and video games	Adolescents	Sleep and mental well-being
	Carson et al., 2016, 71 different countries	Systematic review of 235 studies published 2010–2014 on sedentary behavior and health indicators	School-aged children and youth, mean age: 5–17 years	Self-esteem
Social outcomes	Paulus et al., 2018, Australia, Austria, China, Germany, Iran, Netherlands, Norway, Singapore, South Korea, Spain, Taiwan, UK, USA, multi countries in Europe/Internationally	Systematic review of 252 articles published 1991–2016	Children and adolescents	Online social interactions Real life social interactions
	Mihara and Higuchi, 2017, countries across Europe, North America, and Asia	Systematic review of 37 cross-sectional and 13 longitudinal studies on Internet gaming disorder published up to 2015	National representative samples, students, and online gamer samples	Peer relationships Social network of gamers Education and career attainment Social skills Aggression
	Carson et al., 2016, 71 different countries	Systematic review of 235 studies published 2010–2014 on sedentary behavior and health indicators	School-aged children and youth, mean age: 5–17 years	Conduct/pro-social behavior Academic achievement

**Table 2:** Summary of findings from reviews on the impact of video gaming on health.

**Topic**	**Reference, base location**	**Video gaming measure**	**Outcome measure**	**Impact**
Physical outcomes	Gao et al., 2015, location of studies not reported	Active video games	Physiological outcomes	+
	Mihara and Higuchi, 2017, countries across Europe, North America, and Asia	Internet gaming disorder	Physical pain	–
	Carson et al., 2016, 71 different countries	Video game use	Body composition	Ns
			Blood pressure	Ns
			Cardiometabolic health	Ns
			Cholesterol	Ns
			Cardiorespiratory fitness	–
			Muscular strength/endurance	Ns
	Bilgrami et al., 2017, location of studies not reported	Video game	Obesity	Mix
	Jalink et al., 2014, location of studies not reported	Excessive Nintendo gaming	Injuries	–
Lifestyle behavioral outcomes	Carson et al., 2016, 71 different countries	Video game use	Sedentary behavior	Mix
Cognitive outcomes	Sala et al., 2018, location of studies not reported	Video game players	Cognitive ability	+
	Shams et al., 2015, location of studies not reported	Video games	Some sections of brain structure	+
			Other sections of brain structure	–
	Yao et al., 2017, location of studies not reported	Internet gaming disorders	Some specific neural alterations	+
			Other specific neural alterations	–
Mental and psychological outcomes	Gao et al., 2015, location of studies not reported	Active video games	Psychological outcomes	+
	Mihara and Higuchi, 2017, countries across Europe, North America, and Asia	Internet gaming disorder	Types of games	–
			Personality	–
			Psychological well-being	–
			Mental disorders and sleep	–
	Paulus et al., 2018, Australia, Austria, China, Germany, Iran, Netherlands, Norway, Singapore, South Korea, Spain, Taiwan, UK, US, multiple countries in Europe/internationally	Internet gaming disorder	Psychological distress and reward seeking	–
			Mental disorders	–
			Self-esteem	+
			Self-efficacy	–
	González-Bueso et al., 2018, Australia, Austria, France, Finland, Norway, Germany, South Korea, Singapore, Spain, Sweden, Taiwan, UK, USA	Internet gaming disorder	Depression	–
			Anxiety	–
			ADHD/hyper-activity	–
			Social phobia/social anxiety	–
	Bilgrami et al., 2017, location of studies not reported	New-age technology	Sleep and mental well-being	–
	Carson et al., 2016, 71 different countries	Video game use	Self-esteem	Mix
Social outcomes	Paulus et al., 2018, Australia, Austria, China, Germany, Iran, Netherlands, Norway, Singapore, South Korea, Spain, Taiwan, UK, USA, multiple countries in Europe/internationally	Internet gaming disorder	Online social interactions	+
			Real life social interactions	–
	Mihara and Higuchi, 2017, countries across Europe, North America, and Asia	Internet gaming disorder	Peer relationships	–
			Social network of gamers	+
			Education and career attainment	–
			Social skills	–
			Aggression	–
	Carson et al., 2016, 71 different countries	Video game use	Conduct/pro-social behavior	–
			Academic achievement	Ns

*+: esports have a positive impact on health*.

*–: esports have a negative impact on health*.

*mix: mixed finding, inconclusive of whether esports have a positive or negative impact on health*.

*ns: non-significant findings*.

### Physiological Health Impacts

The impact of video games on physiological health including pain, obesity, injury, and general health indicators (e.g., energy expenditure, VO2 max, metabolic equivalent, body composition, cardiovascular fitness, and sedentary lifestyles), were reviewed by five articles (Jalink et al., 2014; Gao et al., 2015; Carson et al., 2016; Bilgrami et al., 2017; Mihara and Higuchi, 2017).

A systemic review of 234 studies in relation to the health effects of sedentary behavior reported null findings for the association between video game use and body composition, blood pressure, cardiometabolic health, cholesterol, and muscular strength and endurance (Carson et al., 2016). The review reported some evidence of a link between increased video game use and lower levels of cardiorespiratory fitness but inconsistent findings in relation to an association between video game use and sedentary behavior in adolescents. Similarly, a different systemic review of 94 studies examining the health impacts of “new-age technologies” on adolescents reported mixed findings of a link, or lack thereof, between video game play and obesity (Bilgrami et al., 2017). A further systemic review which investigated the relationship between Nintendo video gaming systems and injuries using case studies reported that excessive gaming using traditional controllers with buttons was associated with tendinitis of the extensor of the thumb (Jalink et al., 2014). Active video games (e.g., Wii, Dance Dance Revolution) were linked to better physiological measurements when compared to sedentary behavior, laboratory-based exercise, and field-based physical activity (Gao et al., 2015).

Despite the significant body of literature regarding the physiological health impacts of computer games, there is a clear lack of specific focus on the effects of the competitive gaming aspect of esports on human physiology and physical health, an area of concern which warrants further research. Future research should endeavor to rely on objective measures of physiological health impacts, including sedentary behaviors, rather than self-reported measures.

Three articles examined the effect of video games on cognitive ability, brain structure, and neural alterations, primarily using evidence from imaging studies (Shams et al., 2015; Yao et al., 2017; Sala et al., 2018). Two meta-analyses revealed that video game players outperform non-players in broad measures of cognitive ability. No link was found between video game play and intelligence or reasoning (Sala et al., 2018). However, many of the studies only investigated a single cognitive task, rather than using a model of latent cognitive constructs that considers multiple cognitive measures together. Future studies are required to examine whether effects can be generalized to other non-video game cognitive ability measures.

No study revealed significant definitive findings in relation to the effect of video games on brain structure or the neural alterations seen in individuals with gaming disorder (Shams et al., 2015). It remains unclear whether video games could be used to treat illnesses associated with reduced brain volumes and whether the same neural alterations occur in individuals with different types of gaming disorders. There is thus a clear need for further evidenced-based research regarding the effects of video game play on brain structure and the neural alterations seen in individuals with gaming disorders. As the extant research is predominately comprised of task-based fMRI and VBM studies, future research should draw on other techniques (e.g., diffusion tensor imaging, resting-state fMRI) in order to broaden the knowledge base and accuracy of research in the field.

### Mental Health, Psychological, and Social Impacts

The mental health and psychological impacts of video games and internet gaming disorder, such as general measures of psychological well-being, personality, self-esteem and self-efficacy, sleep, and other specific mental disorders, were reviewed by six articles (Gao et al., 2015; Carson et al., 2016; Bilgrami et al., 2017; Mihara and Higuchi, 2017; González-Bueso et al., 2018; Paulus et al., 2018).

When compared to sedentary behavior, laboratory-based exercise, and field-based physical activities, there is some, albeit limited, evidence that active video games have more positive psychological impacts, particularly in relation to liking and enjoyment, attitudes, intention, self-efficacy, intrinsic motivation, and situational interest (Gao et al., 2015). Studies of video games more generally have not revealed any consistent link between video games and self-esteem (Mihara and Higuchi, 2017). Online social interaction, including through video games, has been found to strengthen meaningful feelings and self-regulation through virtual rank and status, to satisfy otherwise deficient social needs, and to the development of an increased circle of friends (although often with individuals who are addicted to gaming) (Mihara and Higuchi, 2017; Paulus et al., 2018).

Internet gaming disorders have been more closely associated with massively multiplayer online role-playing games, first person shooter games, fighting games, and real time strategy games as compared to other types of games (Mihara and Higuchi, 2017). The disorder has been linked, to varying extents, with higher impulsivity and neuroticism, lower self-esteem, lower self-efficacy, lower life-satisfaction, ADHD, hyperactivity, depression, anxiety, sleeping problems, aggressive tendencies, psychological acceptance of aggression and violence, aggressive behavior, and substance abuse (Bilgrami et al., 2017; Mihara and Higuchi, 2017; González-Bueso et al., 2018; Paulus et al., 2018). Whilst escapism is hypothesized as a potential reason for the link between depression, anxiety and pathological gaming, the direction of the relationship between video games and psychological factors remains unclear (Paulus et al., 2018). On a social level, internet gaming disorders have also been linked to increased loneliness, social phobia, social anxiety, bullying, family problems, and lower levels of education and school grades. The reason for the association between problem gaming and poor family relationships is also unclear (Paulus et al., 2018). Internet gaming disorders have been found to be more prevalent in males and younger age groups (Mihara and Higuchi, 2017; Paulus et al., 2018). There is inconsistent evidence as to whether internet gaming disorders are more common in different geographic regions (Mihara and Higuchi, 2017; Paulus et al., 2018).

Despite its increasing prevalence and the seemingly serious associated health risks, there is a paucity of research regarding internet gaming disorders in the specific context of esports. Although certain types of games have been more closely associated with internet gaming disorder than others, there is a lack of empirical evidence regarding the psychological effects of different types of video games. In light of the significant potential health risks, further research in this area is warranted.

## Discussion

This meta-review has identified the existing evidence regarding the physiological and psychological impacts of online gaming together with the gaps in the existing literature which warrant further research. The majority of the 10 systemic reviews analyzed considered internet gaming disorder in the context of online gaming and esports, but did not investigate in depth the health risks of more casual and intermittent gaming or the watching of esports. Most of the existing research is focused on the physical and cognitive health impacts of video game play and reveals mixed results. There is limited research considering the impacts of video game play on lifestyle factors such as diet and sedentary behavior, though those health risks have been considered in detail in broader contexts.

Our meta-review reveals that video games are likely to have a more positive effect on cognitive ability than on physiological and mental health. Longitudinal and causal studies, including tracking any associations between cognitive skills and stronger educational or employability outcomes, would be a useful research development in this area. Future studies could also examine interventions designed to promote cognitive enhancement observed, through embedded cues in gaming content and gaming structure. Studies reporting impacts of modern competitive gaming design and structure upon cognition, motivation to game, and addiction were lacking, but would be a worthwhile avenue for future research.

Evidence as to the effect of video game play on social factors is mixed, with some studies reporting a link between internet gaming disorders and poor social skills, lower education standards, behavioral problems, and social isolation, and others suggesting that online video game play positively enhances the size of friendship groups and social networks. These mixed results may be attributable to a lack of research attention directed to differentiating long-term gaming from short-term gaming, which might provide differential psychological and health impacts. For example, reviews may have conflated short- and long-term impacts on self-esteem, given that gamers may experience a short-term benefit to self-esteem, that may not persist with heavy and long-term gaming. However, this hypothesis remains to be tested. Whether the positive effect of gaming on social connectivity exists offline remains to be tested and would be a worthwhile subject of future research for the purpose of determining, among other things, whether online gaming could be harnessed to promote connectivity and social cohesion among youth in socially isolated communities.

There is little research on the impacts of gaming on healthy lifestyle behavioral factors, including sedentary behavior, diet, and effective mitigation strategies. Given that obesity and associated adverse health issues are a critical priority for health policy makers globally, and there is evidence of growing sponsorship of esports and in-game product placement by harmful product categories such as alcohol, gambling, and junk food (Kelly and Van der Leij, 2020), research considering the well-being impacts of gaming is needed. Diet and metabolic risks have risen as leading drivers of diseases (Gakidou et al., 2017), but we currently do not have data on whether these risk factors are elevated among the increasing population of competitive online gaming participants. Future research priority areas identified include how esports and competitive video gaming consumption, whether through playing or viewing, affects outcomes across the lifespan, and whether health impacts are robust cross-culturally. Research focused upon esports specifically, encompassing spectating through streaming platforms, in addition to playing, and the immersive nature of esports event attendance, in contrast to video gaming, is limited. While there are studies that have examined the effects of active video games, or exergames, on physical activity-related measures, it remains unclear whether sedentary types of video games are associated with body composition and overall levels of physical activity among players. The broader issue of increased screen engagement has previously been identified as a research priority (Iannotti et al., 2009; Lissak, 2018), highlighting the potential health effects of excessive screen engagement.

The increasing role and power of gaming influencers, some of whom stream to audiences of millions of viewers for hours at a time, warrants examination as it may be a useful vehicle for enhancing positive engagement with online gaming and mitigating adverse health impacts.

Our meta-review reveals a distinct lack of empirical research regarding both the positive and negative health effects of esports and online gaming. This gap exists not only in relation to specific health impacts, but also to more granular examination of gaming frequency, motivation, genre, content, and mode of engagement. Different segments of gamers, such as professional players, streamers, recreational players, and spectators, are likely to have different motivations and impacts from their engagement with esports and online gaming, but these differences remain largely unknown.

Limitations concerning the methodology of this scoping meta-review must be noted. As this review was established as an initial scoping review, our search was limited to a single database and supplementary Google Scholar search, including reviews only, thereby potentially limiting the amount of reviewed evidence. Accordingly, a full systematic review of the physiological and psychological health impacts of esports participation and consumption is warranted.

## Conclusion

This scoping meta-review identified a systematic collation of published reviews concerning the physiological and mental health effects of esports and online gaming. Online gaming has traditionally been perceived as unhealthy due to its sedentary nature (Hilvoorde and Pot, 2016). However, there has been limited empirical research into more specific positive and negative physiological and mental health impacts, particularly in the context of esports. A full systematic review of the health impacts of esports and competitive online gaming, including physical and mental health and social impacts is therefore warranted and would advance the current lack of knowledge on this increasingly critical issue.

## Author Contributions

All authors listed have made a substantial, direct and intellectual contribution to the work, and approved it for publication.

## Conflict of Interest

The authors declare that the research was conducted in the absence of any commercial or financial relationships that could be construed as a potential conflict of interest.
